# TLR4 enhances histamine-mediated pruritus by potentiating TRPV1 activity

**DOI:** 10.1186/s13041-014-0059-9

**Published:** 2014-08-21

**Authors:** Hyunjung Min, Hyunkyoung Lee, Hyoungsub Lim, Yong Ho Jang, Sung Jun Chung, C Justin Lee, Sung Joong Lee

**Affiliations:** 1 Department of Neuroscience and Physiology, Dental Research Institute, School of Dentistry, Seoul National University, Seoul 110-749, Korea; 2 Department of Physiology, College of Medicine, Hanyang University, Seoul 133-791, Republic of Korea; 3 Center for Functional Connectomics, Brain Science Institute, Korea Institute of Science and Technology, Seoul 136-791, Republic of Korea

**Keywords:** Toll-like receptor, Itch, Sensory neurons

## Abstract

**Background:**

Recent studies have indicated that Toll-like receptor 4 (TLR4), a pathogen-recognition receptor that triggers inflammatory signals in innate immune cells, is also expressed on sensory neurons, implicating its putative role in sensory signal transmission. However, the possible function of sensory neuron TLR4 has not yet been formally addressed. In this regard, we investigated the role of TLR4 in itch signal transmission.

**Results:**

TLR4 was expressed on a subpopulation of dorsal root ganglia (DRG) sensory neurons that express TRPV1. In TLR4-knockout mice, histamine-induced itch responses were compromised while TLR4 activation by LPS did not directly elicit an itch response. Histamine-induced intracellular calcium signals and inward currents were comparably reduced in TLR4-deficient sensory neurons. Reduced histamine sensitivity in the TLR4-deficient neurons was accompanied by a decrease in TRPV1 activity. Heterologous expression experiments in HEK293T cells indicated that TLR4 expression enhanced capsaicin-induced intracellular calcium signals and inward currents.

**Conclusions:**

Our data show that TLR4 on sensory neurons enhances histamine-induced itch signal transduction by potentiating TRPV1 activity. The results suggest that TLR4 could be a novel target for the treatment of enhanced itch sensation.

## Background

Chronic itch is a pathological hallmark of atopic dermatitis and other infectious skin diseases. Recent studies on single nucleotide polymorphisms implicate genes encoding pattern-recognition receptors such as CD14 and toll-like receptors (TLRs) in the development and severity of atopic dermatitis (AD) [1],[2]. TLRs are type I transmembrane receptors expressed on innate immune cells that detect pathogen infection or tissue damage. TLR activation of innate immune cells leads to expression of inflammatory mediators including cytokines, chemokines, leukotriene molecules, and histamine [3],[4]. Some of these inflammatory mediators excite sensory neurons and may trigger transmission of itch signals. Therefore, itch sensations observed during skin diseases and infections are generally attributed to sensory neuronal activation by inflammatory mediators released from innate immune cells such as mast cells or keratinocytes [5].

Recently, some pathogen-recognition receptors including TLR3, 4, and 7 were also found to be expressed on sensory neurons [6]. These findings imply that itch signals can also be generated if these TLRs were expressed on itch-specific sensory neurons and activated by pathogen-derived molecules. This possibility is supported by a recent study showing that TLR3 activation on a subpopulation of dorsal root ganglia (DRG) sensory neurons elicits inward currents and action potential [7]. The study found that TLR3 is required for maximal pruritic effects of histamine as well as non-histaminergic pruritogens. In another study, Liu et al. found that TLR7 stimulation by imiquimod, a synthetic TLR7 agonist, directly excites sensory neurons, leading to itch-specific signal generation [8]. However, the same group later reported that TLR7-mediated sensory neuron activation by microRNA elicit pain rather than itch signals [9]. We have also reported the pruritogenic function of imiquimod; however, this was not mediated by TLR7 [10]. Thus, the pruritogenic function of TLR7 on DRG sensory neurons is controversial.

In addition to TLR3 and 7, TLR4 is reported to be expressed in certain DRG and trigeminal ganglia sensory neurons [11]. This finding suggests a role for this receptor in sensory transmission, but this has not been formally studied. Therefore, we investigated the function of sensory neuron TLR4 in itch signal transmission. We found that TLR4 contribute to histamine-induced itch signal transduction at least partly by potentiating TRPV1 activity.

## Results

**Figure 1:**
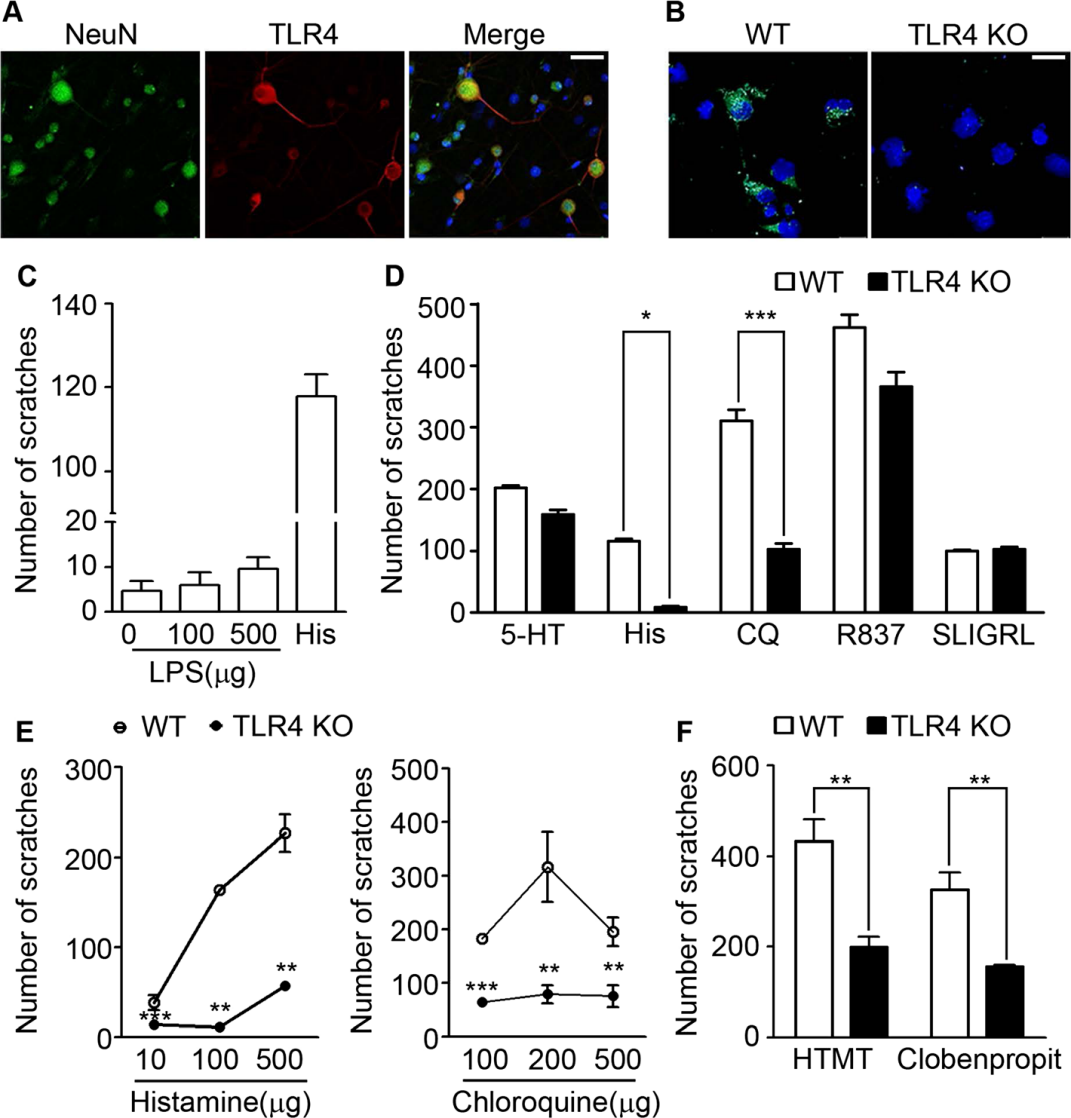
**TLR4 enhances histamine-induced and chloroquine-induced itch sensation. A.** Primary cultured DRG neurons were immunostained with NeuN and TLR4 antibodies. Scale bar, 50 μm. **B.** Primary sensory neurons from WT but not TLR4 KO mice were stained with Alexa488-conjugated LPS. Representative results are shown. Scale bar, 20 μm. **C.** LPS (100 or 500 μg) or histamine (100 μg) was introduced intradermally to WT mice under the nape. Scratching bouts were counted for 30 min (n = 5). LPS at either concentration did not induce significant scratching. **D.** WT or TLR4 KO mice were treated with pruritogens 5-HT 100 nmol, chloroquine (CQ) 200 μg, histamine 100 μg, imiquimod (R837) 200 μg, or SLIGRL 50 μg and scratching bouts were counted for 30 min (n = 6; **p < 0.01, *** *p* < 0.001). **E.** Histamine and chloroquine were introduced to WT and TLR4 KO mice at different doses and scratching bouts were measured (n = 5; **p < 0.01, ***p < 0.001). **F.** Histamine-receptor agonists HTMT (300 nmol) and clobenpropit (100 nmol), were used to treat WT and TLR4 KO mice and scratching bouts were counted (n = 6; ** *p* < 0.01).

We began by checking TLR4 expression in acutely isolated DRG neurons. Immunostaining detected TLR4 in a subpopulation (~21%) of DRG neurons of all sizes (Figure 1A). LPS binding to neuronal TLR4 was confirmed by staining with fluorescently labeled LPS (Figure 1B), with fluorescence signal detected in wild-type (WT) DRG neurons but not in TLR4-deficient neurons. To test if activation of TLR4 on sensory neurons directly triggers itch sensation, we introduced LPS intradermally into the nape of mice and counted the number of scratching bouts for 30 min. In contrast to histamine, administration of up to 500 μg LPS did not significantly enhance the scratch response over the basal level (Figure 1C). These data indicated that TLR4 activation on sensory neurons did not induce an itch signal *per se*. We then tested if TLR4 is involved in itch signal transmission triggered by the pruritogens 5-HT, histamine, chloroquine (CQ), imiquimod (R837), and SLIGRL. While scratching responses seen in TLR4 KO mice after 5-HT, SLIGRL, or imiquimod injection were comparable to responses in WT mice, histamine-induced and chloroquine-induced scratching responses were severely compromised in TLR4 KO mice (Figure 1D and E). The pruritogenic histamine effect is mediated by two histamine receptors, HRH1 and HRH4 [12]. To discern the receptor type involved in the TLR4 effects, we used the HRH1 agonist HTMT and the HRH4 agonist clobenpropit. Both HTMT-induced and clobenpropit-induced scratching responses were comparably reduced as histamine in TLR4 KO mice (Figure 1F). These data show that TLR4 contributes to both HRH1- and HRH4-mediated pruritogenic histamine responses.

**Figure 2:**
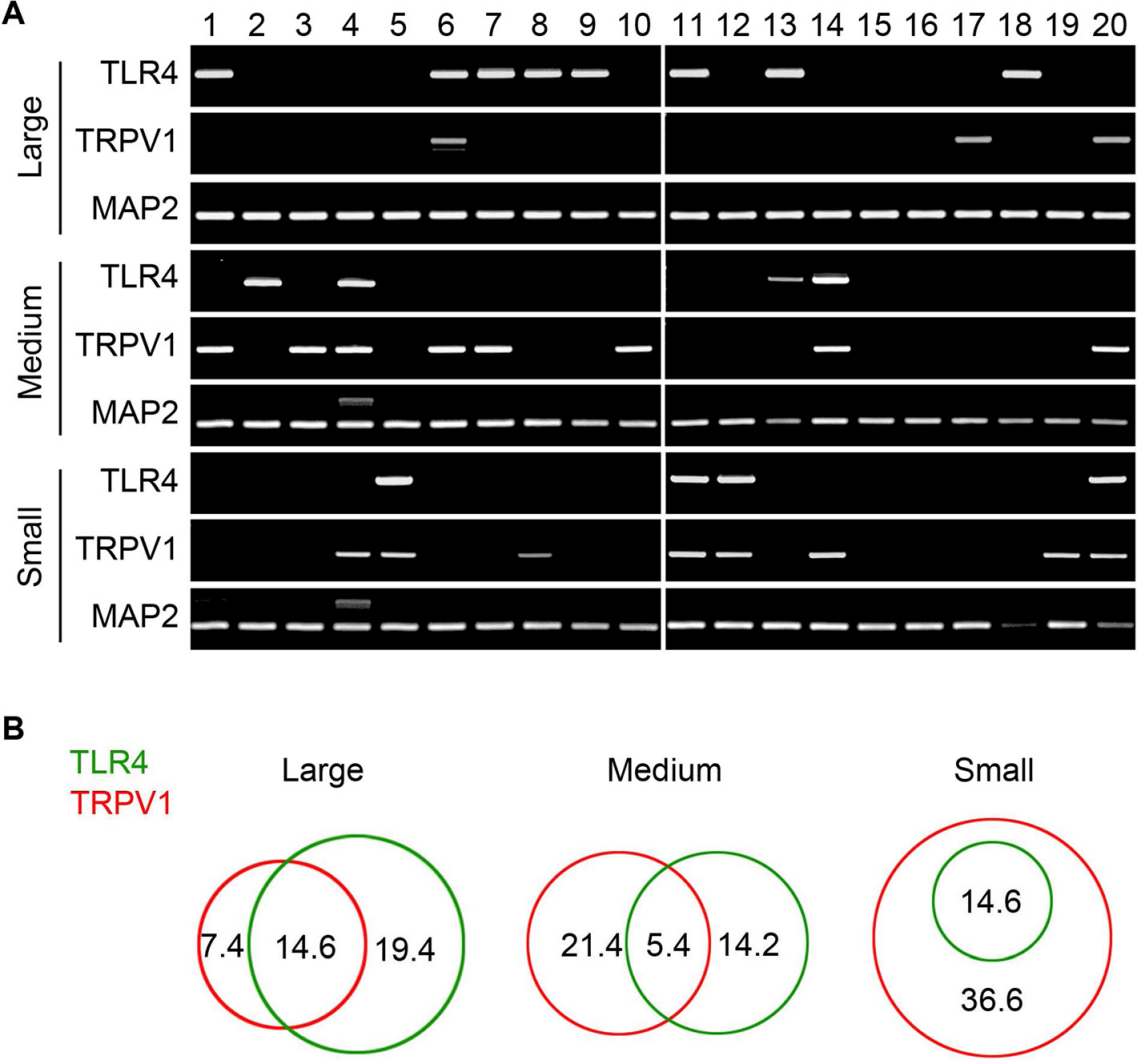
**Characterization of the TLR4-expressing sensory neurons. A**. Expression of TLR4, TRPV1, and MAP2 measured in small (n = 41), medium (n = 56), and large (n = 53) sensory neurons by single-cell RT-PCR. **B**. Profiles of TLR4 and TRPV1 expression in Venn diagram. The percentage of subpopulation is denoted in Venn diagram.

We then analyzed TLR4-expressing sensory neuronal cell types by single-cell RT-PCR. Similar to the immunostaining results, TLR4 transcripts were detected in DRG neurons of all sizes (Figure 2A). TLR4 was expressed in 15% of small neurons (<20 μm), 20% of medium neurons (20 ~ 40 μm), and 34% of large neurons (>40 μm) (Figure 2B). In small neurons, all TLR4+ neurons also expressed TRPV1, while only 28% of TLR4+ medium neurons and 43% of TLR4+ large neurons were TRPV1-positive (Figure 2A and B). Of TRPV1-positive neurons, 29, 20, and 67% cells were TLR4-positive in small-, medium-, and large-sized neurons, respectively. Studies so far indicate that histamine-induced itch signal is generated by small-sized TRPV1+ neurons [13]. Thus, our data suggest that 15% of small DRG neurons that express both TRPV1 and TLR4 might be critical for histamine-induced itch transmission, although TLR4 expression was not restricted to itch-transmitting neurons.

**Figure 3:**
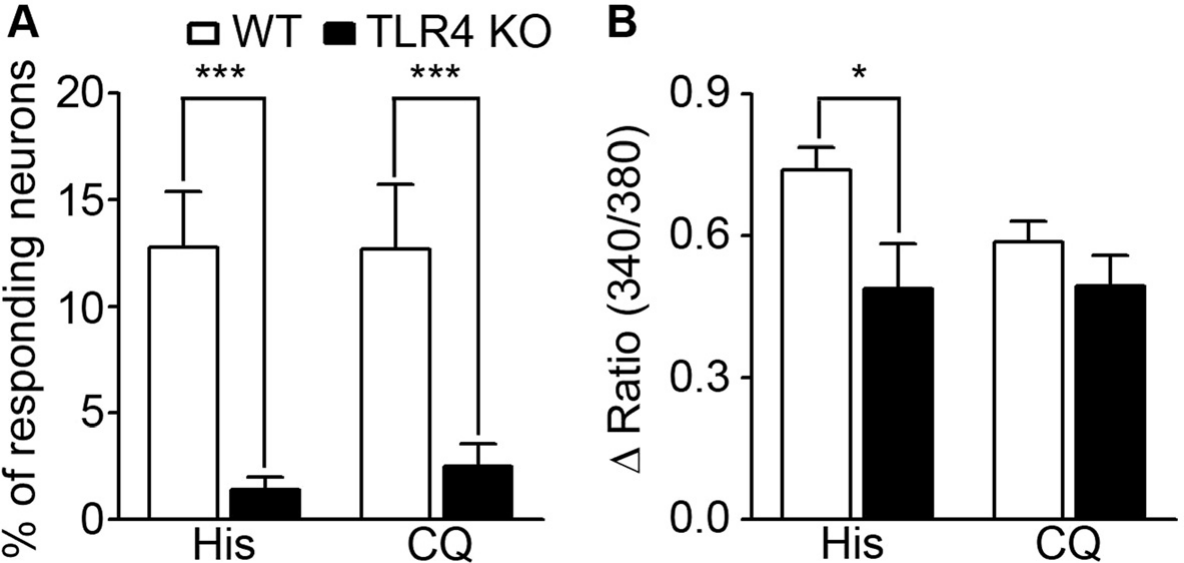
**Histamine-induced and chloroquine-induced intracellular calcium increases were reduced in TLR4 KO neurons. A** and **B**. DRG neurons loaded with Fura2-AM were treated with histamine (10 μg/ml) and chloroquine (CQ, 10 μg/ml) and intracellular calcium level was monitored by calcium imaging. Percentage of responding cells **(A)** and average net increase of [Ca^++^]_i_ (Δ ratio (340 nm/380 nm)) in the responding population **(B)** are in graphs (n = 5, 544 cells measured; *p < 0.005).

Both histamine and chloroquine increase intracellular calcium concentration ([Ca^++^]_i_) in sensory neurons [5],[13]. In primary cultured mouse DRG neurons, histamine treatment increased [Ca^++^]_i_ in 13% of cells, similar to previous results [14]. However, in DRG neurons cultured from TLR4 KO mice, less than 2% of cells responded to histamine treatment (Figure 3A). The average net increase of [Ca^++^]_i_ in the histamine-responsive subpopulation was also reduced by 30% in TLR4-deficient DRG neurons (Figure 3B). The chloroquine-responding cell population was also decreased in TLR4-deficient DRG cells (Figure 3A), although the average [Ca^++^]_i_ increase was not altered (Figure 3B).

**Figure 4:**
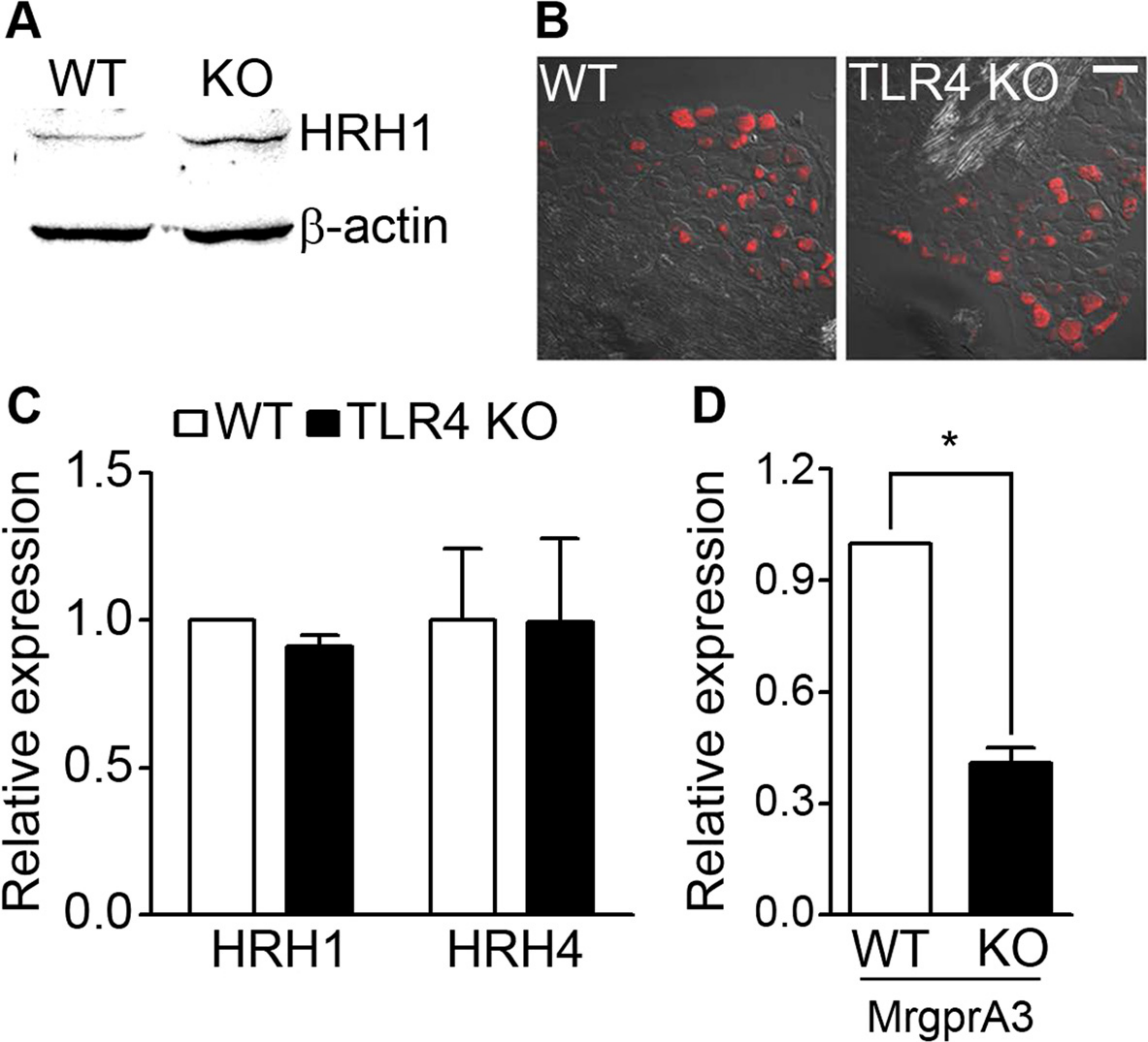
**Histamine receptor expression was not affected by TLR4. A**. DRG neurons from WT and TLR4 KO mice were cultured and HRH1 was measured by western blot. A representative gel is shown (n = 3). **B**. DRG sections from WT and TLR4 KO mice were immunostained with HRH1 antibody. Representative data are shown (n = 3). Scale bar, 50 μm. HRH1-expressing neuronal percentage and size distribution were not noticeably altered in TLR4 KO DRG. **C** and **D**. The mRNA expression of histamine receptors (HRH1 and HRH4) **(C)** and MrgprA3 **(D)** were measured by real-time RT-PCR in WT and TLR4 KO sensory neurons (n = 3). The MrgprA3 expression is significantly reduced in TLR4 KO neurons (*p < 0.005).

To investigate the mechanisms of these results, we tested if TLR4 affected histamine receptor expression in sensory neurons. HRH1 level was not reduced, but was rather increased in TLR4 KO DRG neurons (Figure 4A). The number of HRH1+ neuronal cells as measured by immunohistochemistry was not markedly altered (Figure 4B). Although transcripts for HRH1 and HRH4 were not reduced in TLR4 KO DRGs (Figure 4C), the expression of MrgprA3, a chloroquine receptor, was significantly reduced in TLR4 KO DRG neurons (Figure 4D). These data suggested that the decrease in histamine-induced calcium signals was not due to a reduction in histamine receptor expression levels, although the reduced MrgprA3 expression might contribute to the decreased chloroquine-induced calcium response in TLR4-deficient sensory neurons.

**Figure 5:**
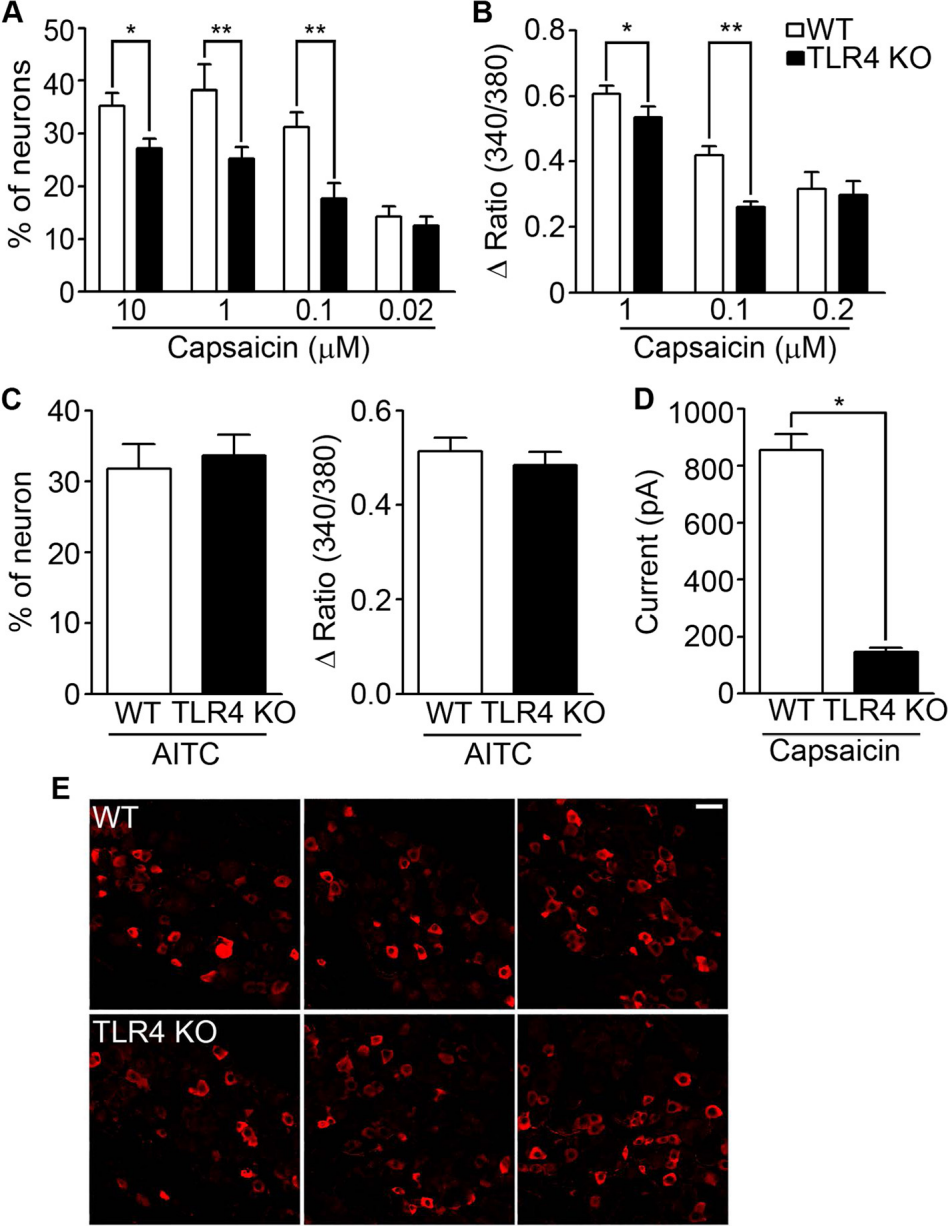
**Capsaicin-induced intracellular calcium signals and inward currents were compromised in TLR4 KO sensory neurons. A** and **B**. DRG neurons from WT and TLR4 KO mice were cultured and treated with capsaicin (0.02-10 μM), and intracellular calcium was monitored by calcium imaging assays. Percentage of responding cells **(A)** and average net increase of [Ca^++^]_i_ (Δ ratio (340 nm/380 nm)) in the responding population **(B)** are shown in graphs (n = 5 with 791, 232, 335, and 215 cells measured; *p < 0.005). Numbers of capsaicin-responding cells and average calcium [Ca^++^]_i_ increase were compromised in TLR4 KO neurons. **C**. TRPA1-dependent calcium signals measured after AITC (1 μM) treatment. TRPA1-mediated calcium signals were not altered in TLR4 KO neurons. **D**. Capsaicin-induced inward currents measured by whole cell patch-clamp recording. Average inward current amplitude in capsaicin-responding TLR4 KO neurons (n = 43; *p < 0.005) was lower than in WT neurons (n = 39; *p < 0.005). **E**. TRPV1 expression was measured by immunohistochemistry using WT and TLR4 KO DRG sections. The percentage of TRPV1-expressing neurons and size distribution were not noticeably altered in TLR4 KO DRG. Representative pictures are shown. Scale bar, 25 μm.

Histamine-induced [Ca^++^]_i_ increase and action potentials in sensory neurons are mediated by TRPV1 channels [13]. Since no difference was detected in histamine receptor levels between WT and TLR4 KO DRG neurons, we tested the possibility that TLR4 affected histamine responsiveness by regulating TRPV1 activity. By measuring capsaicin-induced intracellular calcium signals in sensory neurons (Figure 5A), we found that capsaicin treatment triggered a [Ca^++^]_i_ increase in 35% of WT sensory neurons. The percentage of capsaicin-responding neurons was significantly reduced in TLR4-deficient neurons. The average [Ca^++^]_i_ increase in the capsaicin-responding population was also reduced in the TLR4-deficient neurons compared with WT neurons (Figure 5B). Chloroquine induces [Ca^++^]_i_ increase in the sensory neurons via TRPA1 [5]. When we tested for a TRPA1-induced [Ca^++^]_i_ increase by treating cells with AITC, a synthetic TRPA1 agonist, neither AITC-responding cell numbers nor average [Ca^++^]_i_ increase in AITC-responding neurons was altered in TLR4 KO sensory neurons (Figure 5C). These data showed that TRPV1 activity was specifically affected in TLR4 KO sensory neurons. We then measured inward currents in capsaicin-treated sensory neurons by whole-cell patch clamp recording (Figure 5D). Similar to the results on the capsaicin-induced intracellular calcium signal, the average inward current after capsaicin stimulation was significantly reduced in TLR4 KO neurons compared with WT neurons (Figure 5D). In the meanwhile, neither TRPV1+ cell number nor distribution of TRPV1+ neurons based on cell size was noticeably different in DRGs from TLR4 KO mice compared to WT mice (Figure 5E). Taken together, these data suggested that TLR4 does not affect TRPV1 expression, but rather affects histamine responsiveness by regulating TRPV1 activity in sensory neurons.

**Figure 6:**
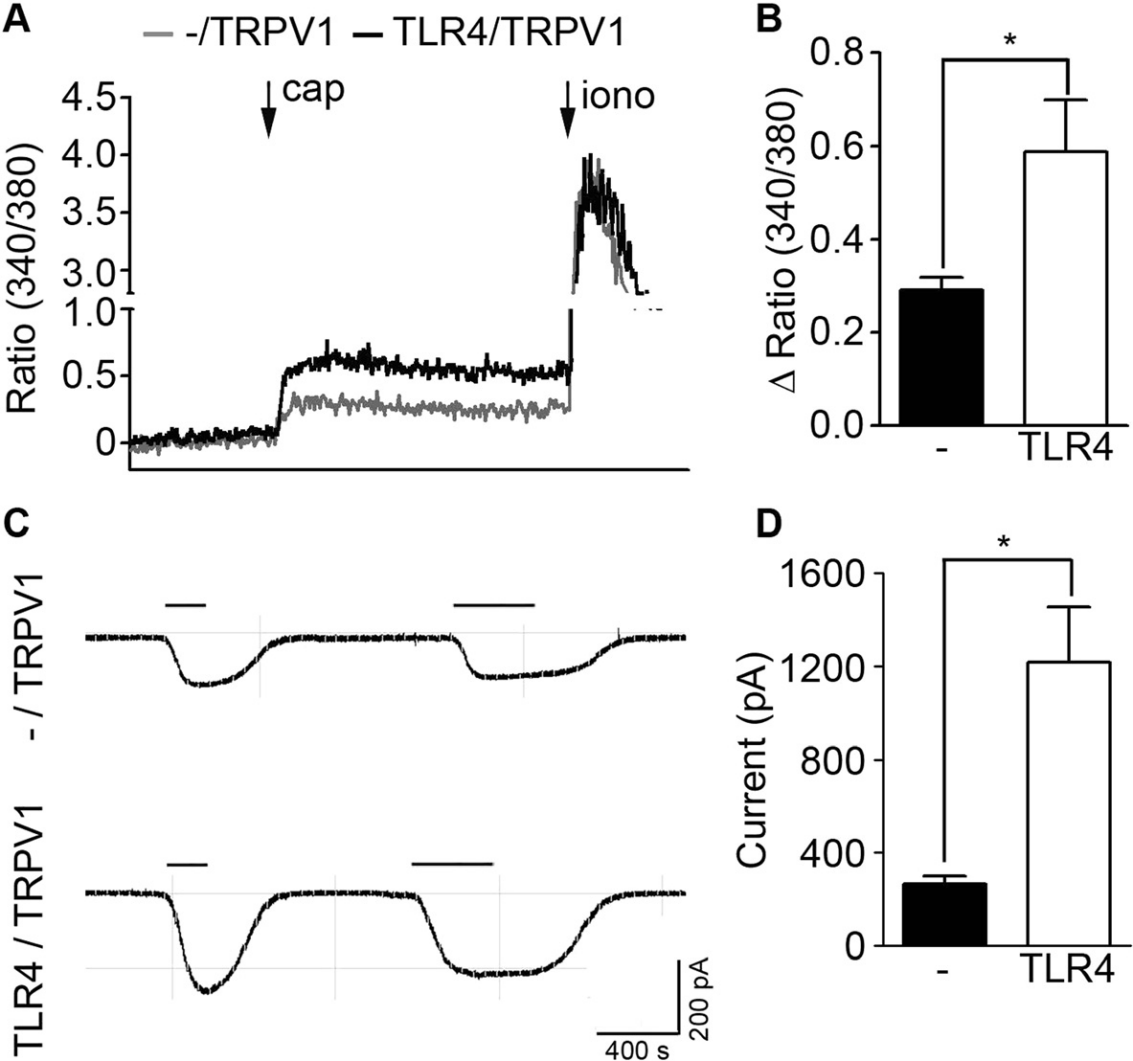
**TLR4 expression enhances TRPV1 activity in HEK293T cells. A**. HEK293T cells transiently overexpressing TRPV1 or TRPV1 plus TLR4 were loaded with Fura2-AM. Cells were treated with capsaicin (10 μM) followed by ionomycin (0.3 μg/ml) and intracellular calcium level was measured by population assay. Representative traces are shown. **B**. Average net increase of [Ca^++^]_i_ (Δ ratio (340 nm/380 nm)) is shown in a graph (n = 6). **C**. Whole cell patch clamp recording was performed using HEK293T cells transiently overexpressing TRPV1 or TRPV1 plus TLR4. Representative traces of capsaicin-induce inward currents are shown. **D**. The average amplitude of the capsaicin-induced inward current in TRPV1-alone or TRPV1-plus-TLR4-expressing HEK293T cells (n = 18; *p < 0.005). The inward current in TLR4-expressing cells is six times the current of TLR4-deficient cells.

To investigate the mechanisms underlying the TRPV1-potentiating effects of TLR4, we adopted heterologous expression system. Capsaicin treatment of TRPV1-overexpressing HEK293T cells increased [Ca^++^]_i_ (Figure 6A). When cells were co-expressed with TRPV1 and TLR4, the net [Ca^++^]_i_ increase rate further enhanced (0.3 vs. 0.6) (Figure 6B). Similarly, capsaicin-induced inward currents in TRPV1-expressing HEK293T cells were greater in cells that were also expressing TLR4 (Figure 6C and D). These data showed that TLR4 expression increased TRPV1 activity in HEK293T cells.

## Discussion

The recent discovery of TLRs on DRG sensory neurons suggests that these pattern-recognition receptors may function in transduction and/or transmission of sensory signals. Studies thus far implicated TLR3 and TLR7 in itch signal transmission. In this study, we have investigated the roles of TLR4 in sensory transmission and found that TLR4 is required for optimal histamine-induced itch signal transduction through regulating TRPV1 activity. Such role of TLR4 in itch signal transduction is distinct from other TLR members. The activation of TLR3 by poly(IC) treatment directly excited sensory neurons and induced a scratch response [7]. However, TLR4 stimulation by LPS did not directly excite sensory neurons or induce significant scratch response. These data suggest that, unlike TLR3, TLR4 signaling alone in sensory neurons does not elicit itch signal. Instead, histamine-induced and chloroquine-induced itch responses were severely compromised in TLR4 KO mice, suggesting TLR4 is required for optimal itch signal transmission stimulated by these pruritogens. Although a reduction in histamine-induced and chloroquine-induced itch responses were also observed in TLR3 KO mice, the mechanisms are different [7]. In our study, in the absence of TLR4, histamine-induced and chloroquine-induced calcium signals in sensory neurons are severely compromised. These data suggest that the itch phenotype we observed in TLR4 KO mice came from itch signal transduction in sensory neurons. However, TLR3 is not required for pruritogen-induced itch signal transduction in the sensory neurons. Rather, TLR3 contributes to synaptic transmission of the itch signal and central sensitization at the spinal cord level. In addition, TLR3 is expressed mainly in small-sized TRPV1+ neurons. However, TLR4 expression was not limited to small-sized neurons but was also detected in large-sized neurons. TLR4 expression was not limited to TRPV1+ neuron population either. These data suggest that, unlike TLR3, TLR4 expression is not limited to itch-specific sensory neurons. Nonetheless, the subpopulation of TRPV1+ neurons that co-express TLR4 is likely to mainly contribute to histamine-induced itch signal transduction. Currently, the involvement of TLR7 in itch signal transmission is under debate. Imiquimod, a synthetic TLR7 agonist, was found to trigger inward currents and action potentials in TLR7+ sensory neurons and induce an itch-specific response [8]. However, it was recently reported that TLR7 activation in sensory neurons elicits pain [9]. We found that the pruritogenic effects of imiquimod are TLR7-independent, but rather it activates sensory neurons by affecting IP3R and potassium channel [10],[15].

In our study, TLR4 regulates histamine-induced and chloroquine-induced itch by distinct mechanisms. TLR4 potentiates histamine responsiveness by increasing TRPV1 basal activity. However, TLR4 does not affect TRPA1 activity. Instead, TLR4 expression increased MrgprA3 expression. Crosstalk between TLR4 and TRPV1 signals has been documented [16]. In this study, TLR4 activation increased capsaicin-induced intracellular calcium signals and inward currents. However, we did not observe significant differences in calcium signals after LPS treatment (data not shown). Our results are consistent with a report that ultrapure LPS, in contrast to standard-grade LPS, fails to induce neuronal excitability [17]. Instead, our data on decreased TRPV1 activity in TLR4 KO neurons indicate that TLR4 expression potentiates TRPV1 activity. It is not clear how TLR4 enhances TRPV1 activity. A recent study shows that TLR7 can enhance TRPA1 activity by direct physical interaction [9]. This suggests a possibility that TLR4 may also enhance TRPV1 activity by direct interaction, which needs to be tested in the future investigation.

TRPV1 is not only associated with itch signals but also involved in pain signals. In our study, we observed capsaicin-induced paw licking was also slightly reduced in TLR4 KO mice (data not shown). This result suggests that the effect of neuronal TLR4 might not be specific to itch signal, but might be involved in pain transmission as well. Of note, nerve-injury induced mechanical allodynia and hyperalgesia are severely compromised in TLR4 KO mice [18]. In this study, TLR4 involvement was interpreted mainly from the perspective of spinal cord microglia activation. Considering our data, it needs to be re-evaluated the putative contribution of sensory neuron TLR4 to the neuropathic pain development.

Itch susceptibility to pruritogens or bacterial/viral infections depends on genetic background. For instance, polymorphisms in genes for the TLR family and associated receptors are implicated in itch-associated diseases such as AD [1]. Our study found a putative mechanism how TLR4 expression affects itch signal transduction. In addition to polymorphisms, TLR4 expression is regulated in diseases such as bacterial infection and tissue damage. In innate immune cells, TLR4 is upregulated by various inflammatory mediators such as TNF-α and IL-1β. Whether sensory neuron TLR4 is also upregulated by these inflammatory mediators is unknown. If it is, inflammatory mediators secreted during the innate immune response in the skin might regulate itch sensitivity by upregulating TLR4 expression in sensory neurons.

## Conclusions

In this study, we have uncovered that TLR4 expression in sensory neurons potentiates histamine-induced itch signal transduction. The histamine-induced scratch response is significantly compromised in TLR4 KO mice. Similarly, histamine-induced intracellular calcium signals and inward currents are reduced TLR4-deficent sensory neurons. As a mechanism for these results, we found that TLR4 expression potentiates histamine-mediated itch by regulating TRPV1 channel activity. Our data suggest that TLR4 could be a novel therapeutic target for treating enhanced itch sensations during inflammatory responses.

## Methods

### Mice

Eight-week-old C57BL6 mice were purchased from Daehan Biolink (Eumsung, Korea). TLR4 KO mice of C57BL6 background were generously provided by Dr. S. Akira (Osaka University, Japan). Mice were housed at 23 ± 2°C with a 12-h light–dark cycle and fed food and water ad libitum. All surgical or experimental procedures were reviewed and approved by the Institutional Animal Care and Use Committee (IACUC) at Seoul National University.

### Behavior study

Scratching behavioral assays were performed as previously described [10]. Briefly, mice were placed individually in transparent cages for at least 30 minutes before assays and received pruritogens or LPS by intradermal injection into the rostral back. Numbers of scratching bouts that included only the hind paw were analyzed at 5-minute intervals for 30 minutes.

### Primary DRG neuron culture

DRGs collected from 8-week-old mice were incubated in Hank’s balanced salt solution (HBSS) (Welgene, Daegu, Korea) containing 0.33 mg/ml papain (Wothington, Lakewood, NJ, USA) and 0.65 mg/ml L-cysteine (Sigma-Aldrich, St. Louis, MO, USA) for 10 minutes at 37°C and then HBSS containing 4 mg/ml collagenase (Roche, Mannheim, Germany) and 5 mg/ml dispase (Invitrogen, Calrsbad, CA, USA) for 10 minutes at 37°C. Samples were washed with DMEM/F12 with 10% (v/v) fetal bovine serum (Invitrogen), 2 mM L-glutamine, 100 U/ml penicillin, 100 μg/ml streptomycin (Welgene). The cell suspension was filtered through a 70-μm cell strainer and cultured in poly-D-lysine (Sigma-Aldrich) and laminin-coated culture dish or glass coverslips (Sigma-Aldrich).

### Cell culture and transfection

HEK293T cells were maintained in DMEM containing 10% FBS, 100 U/ml ampicillin, 100 μg/ml streptomycin, and 2 mM L-glutamine in a humidified incubator containing 5% CO_2_. HEK293T cells were transfected using Effectene transfection reagent (Qiagen, Venlo, Netherlands) according to the manufacturer’s instructions.

### Single-cell RT-PCR

Single-cell RT-PCR was performed as previously described [19]. Briefly, single DRG neuronal cells were collected with a glass micropipette connected to micromanipulator under a microscope and transferred to a PCR tube containing RNase-free water. Digestion with DNase I was performed before reverse transcription for 1 h at 50°C. The cDNA product was used for PCR in 50 μl of PCR buffer containing 0.2 mM dNTPs, 0.2 μM outer primers, 5 μl RT product, and 0.2 μl platinum *Taq* DNA polymerase (Invitrogen). The protocol was 5 min at 95°C followed by 40 cycles of 40 s at 95°C, 40 s at 60°C, and 40 s at 72°C. Reactions were completed with 7 min of final elongation. The second round of amplification was in reaction buffer (20 μl) containing 0.2 mM dNTPs, 0.2 μM inner primers, 5 μl first-round PCR product, and 0.1 μl platinum *Taq* DNA polymerase following the same amplification procedure as the first round. The following primers were used: TLR4 outer forward 5′- ATC TGA GCT TCA ACC CCT TG-3′; TLR4 outer reverse 5′- AAT TCC CTG AAA GGC TTG GT-3′; TLR4 inner forward 5′- TCA GAA CTT CAG TGG CTG GA-3′; TLR4 inner reverse 5′- TTG ACT TGT GGA TTT TCA CG-3′; TRPV1 outer forward 5′- CAT GCT CAT TGC TCT CAT GG-3′; TRPV1 outer reverse 5′- AAC CAG GGC AAA GTT CTT CC-3′; TRPV1 inner forward 5′- CAT GGG CGA GAC TGT CAA C-3′; TRPV1 inner reverse 5′- CTG GGT CCT CGT TGA TGA TG-3′; MAP2 outer forward 5′- GAA GAG TTC CAA GGC CCA CTT-3′ MAP2 outer reverse 5′- GCC TGA AAT TTG CCT TTT CC-3′; MAP2 inner forward 5′- CCT GTG CAA TTC CAG CTC AGT-3′; MAP2 inner reverse 5′- CCC CCA TGT GGC ATG AAA TAT3′.

### Immunofluorescence

For DRG staining, adult mice were anesthetized with urethane and perfused with 4% paraformaldehyde and DRGs were collected. DRGs were incubated in 4% paraformaldehyde, cryoprotected in 30% sucrose, and frozen in OCT. DRG sections (12 μm) were obtained by cryostat and mounted on collagen-coated slides. Sections were stained with mouse anti-histamine receptor H1 (Santa Cruz, Dallas, TX, USA) or anti-TRPV1 (Santa Cruz) then incubated for 1 h with Cy3-conjugated secondary antibodies (1:200; Jackson ImmunoResearch, West Groove, PA, USA), and coverslipped with VectaShield medium (Vector Labs, Burlingame, CA, USA).

For immunocytochemistry, DRG neuronal cells or transfected HEK293T cells were seeded onto poly-D-lysine (PDL)-coated coverglass. Cells were fixed in 4% PFA in 0.1 M PBS (pH 7.4) for 15 min. After rinsing in 0.1 M PBS, cells were blocked with 0.1 M PBS containing 5% normal goat serum, 5% fetal bovine serum, 2% bovine serum albumin, and 0.1% Triton X-100 for 1 h at RT. Cells were incubated overnight at 4°C with mouse anti-NeuN (1:1000; Millipore, Billerica, MA, USA), rabbit anti-TLR4 (1:100; Santa Cruz), or mouse anti-HA (1:1000; Cell signaling, Danvers, MA, USA) antibody. Cells were incubated for 1 h at RT with FITC- or Cy3-conjugated secondary antibody and mounted with VectaShield medium.

For live cell staining, DRG neurons were seeded on PDL-coated coverglasses and incubated with 5 μg/ml Alexa Fluor 488-conjugated LPS from *Escherichia coli* O55:B5 (Molecular Probes, Eugene, OR, USA) for 1 h at 37°C. After rinsing in 0.1 M PBS, coverglasses were mounted with VectaShield medium. Images were captured using a confocal laser scanning microscopy (LSM7 PASCAL; Carl Zeiss, Jena, Germany).

### Real-time RT-PCR

Real-time RT-PCR was performed using SYBR Green PCR Master Mix (ABI, Warrington, UK) as described previously [20]. PCR was performed in duplicate in a total volume of 10 μl containing 10 pM primer, 4 μl cDNA, and 5 μl SYBR Green PCR Master Mix. The mRNA levels of each target gene were normalized to GAPDH mRNA. Fold-induction was calculated using the 2^-∆∆CT^ method as previously described [21]. All real-time RT-PCR experiments were performed at least three times and are presented as mean ± SEM unless otherwise noted.

Primers for real-time RT-PCR were MrgprA3 forward: 5’-CGA CAA TGA CAC CCA CAA CAA-3’; MrgprA3 reverse: 5’-GGA AGC CAA GGA GCC AGA AC-3’; histamine receptor H1 forward: 5’-GGG AAA GGG AAA CAG TCA CA-3’; histamine receptor H1 reverse: 5’-ACT GTC GAT CCA CCA AGG TC-3’; histamine receptor H4 forward: 5’-GGA AGC TAG CCA GGT CAC T-3’; histamine receptor H4 reverse: 5’-CCG TTC TGG AAG TTG AA-3’; TRPV1 forward: 5’-AAG GCT CTA TGA TCG CAG GA-3’; TRPV1 reverse: 5’-CAG ATT GAG CAT GGC TTT GA-3’; GAPDH forward: 5’-AGG TCA TCC AGC TGA ACG-3’; GAPDH reverse: 5’-CAC CCT GTT GCT GTA GCC GTA T-3’.

### Calcium assays

Calcium response in DRG neurons was measured by single-cell calcium imaging using Fura-2 AM (Invitrogen). Cells were plated on PDL-coated coverglasses and incubated overnight. Cells were incubated for 50 min at RT with 2 μM Fura-2 AM in HBSS containing 25 mM HEPES (pH 7.5) and washed with HBSS-HEPES twice before assays. A baseline reading was taken for 60 s before addition of histamine, chloroquine or capsaicin. After treatment, to test cell viability, 100 mM KCl was added. Intracellular calcium levels were measured by digital video microfluorometry with an intensified charge-coupled device camera (CasCade, Roper Scientific, Trenton, NJ, USA) coupled to a microscope and analyzed with MetaFluor software (Universal Imaging Corp., Downington, PA, USA). Fura-2 AM excitation wavelengths were selected by a Lambda DG-4 monochromator wavelength changer (Shutter Instrument, Novato, CA, USA).

To determine intracellular calcium levels in HEK293T cell population, cells were detached from plates 24 h after transfection and stained with 2 μM Fura-2 AM in Locke’s solution (154 mM NaCl, 5.6 mM KCl, 1.2 mM MgCl_2_, 2.2 mM CaCl_2_, 5.0 mM HEPES, 10 mM glucose, pH 7.4) for 50 min at 37°C. Cells were then washed twice with Locke’s solution and suspended at 1 × 10^6^ cells/ml for assays. Intracellular calcium levels were monitored with dual excitation at 340 and 380 nm and emission at 500 nm by a spectrofluorophotometer (Shimadzu RF-5301-PC, Shimadzu, Kyoto, Japan). The ratio of emission after 340 nm and 380 nm excitation (340 nm/380 nm) was used for index of intracellular calcium concentration ([Ca^++^]_i._). The net change in [Ca^++^]_i_ upon drug treatment (Δ ratio (340 nm/38 nm)) was calculated by subtracting basal [Ca^++^]_i_ from the peak [Ca^++^]_i_ achieved after exposure to the drug.

### Whole cell patch-clamp recording

Cells were plated on PDL-coated cover glasses and incubated in medium for at least 6 h. DRG neuron recordings were performed in HEPES buffer (10 mM HEPES, 150 mM NaCl, 10 mM KCl, 2 mM MgCl_2_, 5.5 mM glucose, and 22 mM sucrose, pH 7.4). Under an upright microscope, whole-cell patch recordings were obtained from acute-isolated DRG neurons in voltage-clamp mode and switched current-clamp configuration for recordings with an Axopatch 700B (Molecular Devices, Sunnyvale, CA, USA). Pipette resistance ranged from 3 to 6 MΩ. The internal solution was 140 mM K-gluconate, 10 mM HEPES, 7 mM NaCl, 4 mM Mg-ATP, and 0.3 mM Na3-GTP, pH 7.4. Data were analyzed and plotted with pClamp (Molecular Devices).

### Western blot assay

For western blots, protein samples were separated using 10% SDS-PAGE and transferred to nitrocellulose membranes. After blocking with 5% nonfat dry milk in TBST (20 mM Tris, pH 7.4, 0.1% Tween 20, 150 mM NaCl), membranes were incubated with rabbit anti-histamine receptor H1 (1:500, Santa Cruz), or mouse anti-β-actin (1:5000, Sigma-Aldrich) antibodies. Proteins were detected with horseradish peroxidase conjugated secondary antibodies using West Save Gold western blot detection kit (Ab Frontier, Seoul, Korea). Signal was visualized by MicroChemi (DNR Bio-imaging Systems, Jerusalem, Israel).

### Statistical analysis

All data are presented as mean value with SEM. Differences between groups were determined by one-way ANOVA with Tukey’s multiple comparison test or Student’s *t*-test. Differences were considered significant when *p* was less than 0.05.

## Abbreviations

5-HT: 5-hydrixytryptamine

AD: Atopic dermatitis

CD: Cluster of differentiation

CQ: Chloroquine

DRG: Dorsal root ganglia

GAPDH: Glyceraldehyde 3- phosphate dehydrogenase

HBSS: Hank’s balanced salt solution

HRH1: Histamine receptor H1

HRH4: Histamine receptor H4

IL-1β: Interleukine-1β

KO: Knock out

LPS: Lipopolysaccharide

MrgprA3: MAS-related G protein-coupled receptor family, member A3

R837: Imiquimod

TLR4: Toll-like receptor 4

TNF-α: Tumor necrosis factor-α

TRPA1: Transient receptor potential cation channel, subfamily A, member 1

TRPV1: Transient receptor potential cation channel, subfamily V, member 1

## Competing interests

The authors do not have any financial or non-financial competing interests.

## Authors’ contributions

HJ carried out behavior study, primary DRG neuron culture, real-time RT-PCR and calcium assay. HL performed single-cell RT-PCR and immunofluorescence staining with primary DRG neuron. HL performed DRG immunofluorescence staining. YHJ contributed to behavior study. SJC and CJL carried out whole cell patch-clamp recording. SJL designed and supervised all the experiments and analyzed data. All authors read and approved the final manuscript.
